# Genetic profiling of rat gliomas and cardiac schwannomas from life-time radiofrequency radiation exposure study using a targeted next-generation sequencing gene panel

**DOI:** 10.1371/journal.pone.0296699

**Published:** 2024-01-17

**Authors:** Ashley M. Brooks, Andrea Vornoli, Ramesh C. Kovi, Thai Vu T. Ton, Miaofei Xu, Ahmed Mashal, Eva Tibaldi, Federica Gnudi, Jian-Liang Li, Robert C. Sills, John R. Bucher, Daniele Mandrioli, Fiorella Belpoggi, Arun R. Pandiri

**Affiliations:** 1 Integrative Bioinformatics Support Group, Division of Intramural Research, National Institute of Environmental Health Sciences, Research Triangle Park, North Carolina, United States of America; 2 Comparative and Molecular Pathogenesis Branch, Division of Translational Toxicology, National Institute of Environmental Health Sciences, Research Triangle Park, North Carolina, United States of America; 3 Cesare Maltoni Cancer Research Center, Ramazzini Institute, Bologna, Italy; 4 Experimental Pathology Laboratories, Inc., Research Triangle Park, North Carolina, United States of America; University of Michigan Medical School, UNITED STATES

## Abstract

The cancer hazard associated with lifetime exposure to radiofrequency radiation (RFR) was examined in Sprague Dawley (SD) rats at the Ramazzini Institute (RI), Italy. There were increased incidences of gliomas and cardiac schwannomas. The translational relevance of these rare rat tumors for human disease is poorly understood. We examined the genetic alterations in RFR-derived rat tumors through molecular characterization of important cancer genes relevant for human gliomagenesis. A targeted next-generation sequencing (NGS) panel was designed for rats based on the top 23 orthologous human glioma-related genes. Single-nucleotide variants (SNVs) and small insertion and deletions (indels) were characterized in the rat gliomas and cardiac schwannomas. Translational relevance of these genetic alterations in rat tumors to human disease was determined through comparison with the Catalogue of Somatic Mutations in Cancer (COSMIC) database. These data suggest that rat gliomas resulting from life-time exposure to RFR histologically resemble low grade human gliomas but surprisingly no mutations were detected in rat gliomas that had homology to the human *IDH1* p.R132 or *IDH2* p.R172 suggesting that rat gliomas are primarily wild-type for IDH hotspot mutations implicated in human gliomas. The rat gliomas appear to share some genetic alterations with *IDH1* wildtype human gliomas and rat cardiac schwannomas also harbor mutations in some of the queried cancer genes. These data demonstrate that targeted NGS panels based on tumor specific orthologous human cancer driver genes are an important tool to examine the translational relevance of rodent tumors resulting from chronic/life-time rodent bioassays.

## Introduction

Gliomas are the most common primary brain tumors in humans and are classified based on histological and molecular features defined by the WHO criteria [1]. Human brain tumors with diverse morphological features appear to share a limited set of genetic alterations associated with mutations in isocitrate dehydrogenase 1 and 2 (*IDH1/IDH2*) genes along with 1p/19q co-deletion and mutations in telomerase transcriptase (*TERT*) promoter, *Tp53*, and *Atrx* genes. Overall, approximately 80% of adult grade II/III gliomas and secondary glioblastoma multiforme (GBM) harbor mutations at either Arg132 of *IDH1* or Arg172 of *IDH2*, whereas point mutations in the *TERT* promoter occurred in ~75% of GBM [2–5]. These distinct genetic changes have greater prognostic significance than diagnoses based on morphological features alone.

Rat brain tumors (gliomas) share several morphological features with human brain tumors, such as astrocytomas, oligodendrogliomas and mixed glial cell tumors. Rat brain tumors arising spontaneously or due to chronic chemical/agent exposure are classified mainly based on distribution and morphological features of the tumor cells, and to a much lesser extent on immunohistochemical features. Most of the rat brain tumors have minimal expression of glial fibrillary acidic protein (GFAP) although the morphology is comparable to that of human astrocytomas [6–8]. The International Harmonization of Nomenclature and Diagnostic Criteria (INHAND) working group recommended the use of ‘malignant glioma or glioma NOS (not otherwise specified) for rat brain glial tumors due to the difficulty in confirming the cell type and due to cellular heterogeneity [9]. The diagnosis of ‘malignant glioma or glioma NOS’ is convenient to summarize the CNS neoplastic lesions in a rodent carcinogenicity study especially due to their rarity and low incidences in any particular study. However, if compared to human glioma features and terminology, the rat gliomas resemble “diffuse gliomas” and majority of the rat gliomas may be considered low grade based on cellularity, anaplasia, necrosis, and microvascular proliferation. In contrast to human gliomas, information on molecular alterations in rat tumors in very limited due to the significant rarity of these lesions in rodent carcinogenicity studies. Treatment-related brain tumor incidences in rodents are rare in chemical carcinogenicity studies conducted by the US National Toxicology Program (NTP) or elsewhere. In the NTP rodent carcinogenicity studies, less than 2% of the studies had any evidence (clear, some or equivocal) of treatment related brain tumors and only 4/600 distinct exposures resulted in clear or some evidence of carcinogenic activity in the brain [10]. The incidences of spontaneous brain tumors in control rats ranged from 0–2% (NTP historical controls 2013 & 2019). Due to the rare incidences of these tumors, lack of tissue collection designed for molecular studies, and the resulting lack of genomic data, it is a challenge to compare the molecular alterations in chemically induced rat gliomas with human brain cancers. Similarly, cardiac schwannomas are very rare in both rats and humans, and the translational relevance of these tumors in rats remains to be understood, as well.

In a preliminary study (unpublished) in our laboratory, mutation profiling by Sanger sequencing of hot-spot regions of four glioma-related genes (*Idh1*, *Idh2*, *Braf*, *and Egfr*) identified point mutations in *Egfr* gene in 55% (6/11) of the gliomas from chemical-exposed HSD: Sprague Dawley^®^ SD rats. Surprisingly, no mutations were detected in *Idh1*, *Idh2* and *Braf* genes that matched to orthologous hot spot mutations in human gliomas and no mutations were observed in spontaneous gliomas (0/4, data not presented). These findings underscore our lack of knowledge regarding genetic alterations in rat gliomagenesis.

Recently, the NTP tested the carcinogenic potential of radio frequency radiation (RFR) at a frequency of 900 MHz in two modulations used for cellular telephone communications (Global System for Mobile communications (GSM) or Code Division Multiple Access (CDMA). Male and female Sprague Dawley (Hsd:Sprague Dawley SD) rats were exposed to time-averaged whole-body specific absorption rates of GSM- or CDMA-modulated RFR at 900 MHz in utero, during lactation, and after weaning for 28 days or 2 years. At the end of 2 years, exposure related increases in the incidences of gliomas and cardiac schwannomas were observed in male rats with both GSM and CDMA modulations [11]. The Ramazzini institute (RI) in Bologna, Italy also conducted RFR studies in Sprague Dawley rats exposed from prenatal life until natural death to a 1.8 GHz GSM (far field). These studies also showed an increase in the incidences of gliomas and cardiac schwannomas [12]. As discussed earlier, due to the lack of molecular data on these rat tumors, their translational relevance to human health remains to be understood. The rat tissues in the NTP studies were fixed in formalin for prolonged duration and as a result the DNA was significantly degraded. Fortunately, the alcohol-based tissue fixation protocol at RI enabled extraction of higher quality DNA and allowed next generation sequencing studies on tumors from RFR exposures [13].

In this study, we examined archival rat gliomas arising spontaneously or due to life-time exposure to RFR at the RI using a custom designed amplification-based targeted next generation sequencing panel for rat gliomas based on 23 human glioma-related genes to understand the translational relevance of these tumors to human health. Furthermore, we also used this gene panel to investigate rat cardiac schwannomas, a rare tumor originating from the nerve sheath, that developed spontaneously or due to life-time exposure to RFR in rats. This is the first study demonstrating genetic alterations in rat tumors resulting from RFR exposures and provides information on the translational relevance of these rat tumors human health.

## Materials and methods

### Radiofrequency radiation exposure and rat brain tissue samples

Tissue samples for this retrospective study were obtained from animal studies previously conducted at the Ramazzini Institute to assess the carcinogenic hazard related to chronic exposure to radio frequency emissions representative of base station environmental exposure [12]. A 1.8 GHz base station radiation emission system that is similar to GSM cellular telephone base station was used for whole body exposure for the lifetime of Sprague-Dawley (SD) rats as previously described [12]. All the animals received standard feed *ad libitum* provided by Laboratorio Dottori Piccioni (Milan, Italy) and tap water. The animal experiments were conducted in accordance with the Italian law regulating the use of animals for experimental and other scientific purposes [14] as well as the Ramazzini Institute’s animal care and use committee guidelines [12]. Four groups of 817, 811, 411, 409 male and female SD rats were respectively subjected to a 1.8 GHz GSM of 0, 5, 25, 50 V/m (that correspond to a specific absorption rate (SAR) of 0, 1.001, 0.03, 0.1 W/Kg) with a whole-body exposure for 19 continuous hours per day, 7 days a week. SD rat tissues (gliomas = 12 and cardiac schwannomas = 9 from life-time RFR exposure; interim [1 year] sacrificed non-tumor brain tissues from RFR exposed rats [e.g. Exposed Non-tumor Tissues, “ENTs”] = 30; spontaneous gliomas = 2) from the RI-RFR cancer bioassay were examined in this study (S1 and S2 Tables). We have also included unexposed, age-matched (2 year) controls for brain (n = 10), heart (n = 9) and one-year interim sacrificed rats (n = 10). Matched genomic controls for all animals were taken from kidney tissues. At necropsy after life-time exposures, tissues were fixed in 70% alcohol, processed routinely, embedded in paraffin (EFPE) and 5 μm thick sections were cut and stained with hematoxylin and eosin (H&E). Sections of brain tissues from interim sacrifice animals were flash frozen in liquid nitrogen at the time of necropsy for molecular analysis.

#### Nucleic acid isolation

All tumors were reviewed by a committee of board-certified veterinary pathologists to confirm the histomorphological findings. The selected tissues were ensured to contain more than 90% viable tumor tissue and lacked large areas of hemorrhage, necrosis or autolysis. The tissues were then grossly dissected from three to five 10 *μ*m unstained histological sections. Total genomic DNA was isolated from each specimen using the Nucleospin Tissue XS kit^®^ (Macherey-Nagel, Bethlehem, PA, USA). Concentration and quality of the DNA was assessed by Qubit 3.0 fluorimeter using the dsDNA HS assay kit (ThermoFisher Scientific, Waltham, MA, USA) and TapeStation 4200 (Agilent Technologies, Santa Clara, CA, USA), respectively.

#### Designing targeted next generation sequencing gene panel

A targeted next generation sequencing (NGS) panel of 23 glioma-related genes was designed based on curated genetic mutation data from the COSMIC database and published literature representing more than 2000 human glial tumors [5, 15]. The panel was constructed to target all coding regions for 13 genes and known hotspot exons for an additional 10 genes (Table 1). Probes were designed against a 1 kb region upstream of the *Tert* gene transcription start site, select exons for the additional nine hotspot genes and exons, exon-intron boundaries, 5’UTR and 3’UTR for the remaining full length targeted genes (Table 1).

**Table 1:** Cancer panel design listing targeted genes and exons. Details of exon amplification success after probe design are included.

Gene	Target	Regions Amplified	No Amplification
*Arid1a*	All coding exons	All coding exons	.
*Atrx*	All coding exons	Exons 17–35	Exons 1–16
*Cdkn2a*	All coding exons	Exons 1–5	Exons 6–7
*Cic*	All coding exons	All coding exons	.
*Egfr*	All coding exons	All coding exons	.
*Fubp1*	All coding exons	Exons 15–18	Exons 1–14
*Nf1*	All coding exons	Exons 1–18, 20–52,54–61	Exons 19,53
*Pdgfra*	All coding exons	Exons 1–5,8–23	Exons 6–7
*Pik3ca*	All coding exons	Exons 6–20	Exons 1–5
*Pten*	All coding exons	1–3, 5–9	Exons 4
*Rb1*	All coding exons	Exons 1–15, 17–27	Exons 16
*Setd2*	All coding exons	Exons 1–4,6–21	Exons 5
*Tp53*	All coding exons^1^	Exons 2–10	.
*Braf*	Exon 18	Exon 18	.
*Chek2*	Exon 9–11, 13–14	Exon 9–14	.
*Erbb2*	Exons 2–3, 6–9, 19–20	Exons 2–3, 6–9	Exons 19–20
*Hif1a*	Exons 1–6	Exon 6–7	Exons 1–5
*Hras*	Exons 2–3	Exons 2–3	.
*Idh1*	Exon 3	Exon 3	.
*Idh2*	Exon 4	Exon 4	.
*Kras*	Exons 1–2	Exons 1–2	.
*Notch1*	Exons 2–9	Exons 2–9	.
*Tert*	500 upstream TSS	500 upstream TSS	.

#### Library preparation and sequencing

A total of 100 ng input genomic DNA was used for library construction using Illumina TruSeq Custom Amplicon Low Input Kit (Illumina, San Diego, CA, USA). The libraries for the tumors, exposed-non-tumor (ENT) samples and the age-matched genomic controls were prepared following the manufacturer’s instructions, starting with hybridizing the oligo pool, extend and ligate the bound oligos, amplify the libraries with 28 PCR cycles. After library preparation, fragment analysis and quantification were performed with the TapeStation 4200 (Agilent Technologies, Santa Clara, CA, USA) and Qubit 3.0 fluorimeter using the dsDNA HS assay kit (ThermoFisher Scientific, Waltham, MA, USA), respectively. Paired-end or single-end 150 base pair (bp) libraries for rat brain and cardiac tumors, interim sacrificed exposed-non-tumor (ENT) tissues, and age-matched unexposed and kidney control samples were sequenced on Illumina NextSeq (paired-end) and NovaSeq (single-end) platforms following manufacturer’s protocols (Illumina, San Diego, CA, USA).

#### Targeted next generation sequencing data analysis

Sequencing read quality was assessed with the FastQC software [16]. Trim-Galore v. 0.4.4 was used to trim Illumina adapters and discard reads with phred quality score < 30 and length less than 50 bp after trimming. Trimmed reads were aligned to the Rn6 reference genome with Bowtie2 v.2.3.0 [17]. Reads with a mapping quality score (MapQ) < 30 were discarded with the *samtools view* function [18].

**Fig 1:**
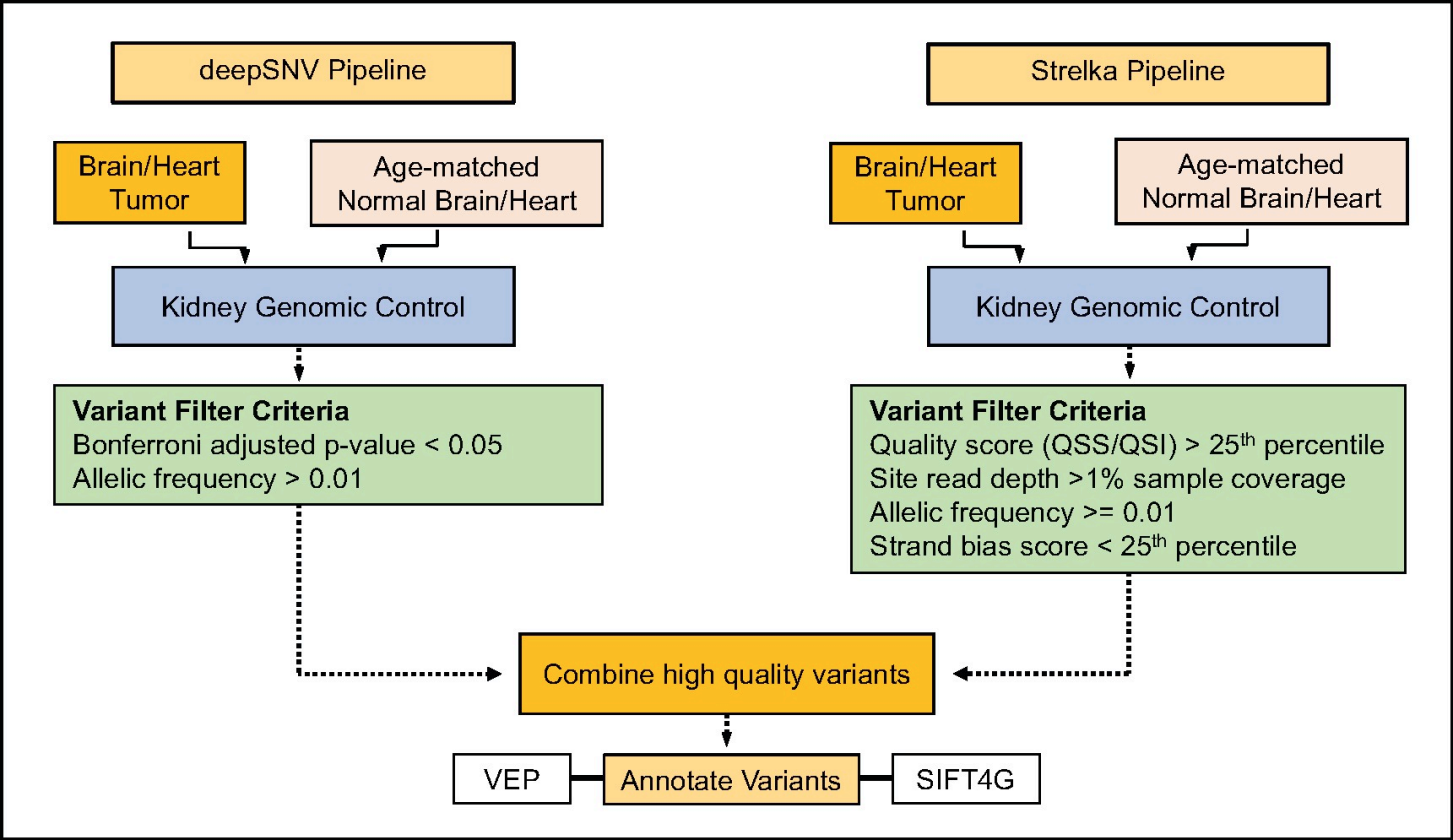
NGS panel data analysis pipeline.

Strelka2 and deepSNV were used to call raw variants in tumor (Fig 1), ENT and age matched control samples with matched kidney tissue as the matched genomic control [19]. Strelka2 was run using default parameters with flags set to—disableEVS and–targeted—callRegions. For deepSNV, the site-specific background error rate was estimated with the beta-binomial model and the Bonferroni method was applied to p-values to control the family wise error rate.

Strelka2 variant filter criteria required site read depth >1% sample coverage, quality score (QSS/QSI) > 25^th^ percentile, strand bias score < 25^th^ percentile and allele frequency > = 0.01. Filter criteria for deepSNV variants required Bonferroni corrected p-value < 0.05 and allele frequency > = 0.01. Variants remaining in the control sets after filtering were combined and subsequently removed from the tumor and ENT variants. The final high confidence variant set was comprised of SNVs and small indels that were shared between filtered Strelka2 and deepSNV variants for each sample.

Variants were annotated with the Ensembl Variant Effect Predictor (VEP) and SIFT 4G [20, 21]). The COSMIC v. 94 database was queried for known human variants with homology to the high confidence rat variants (cancer.sanger.ac.uk; [22]).

## Results

### Comparative pathology of rat tumors

**Fig 2:**
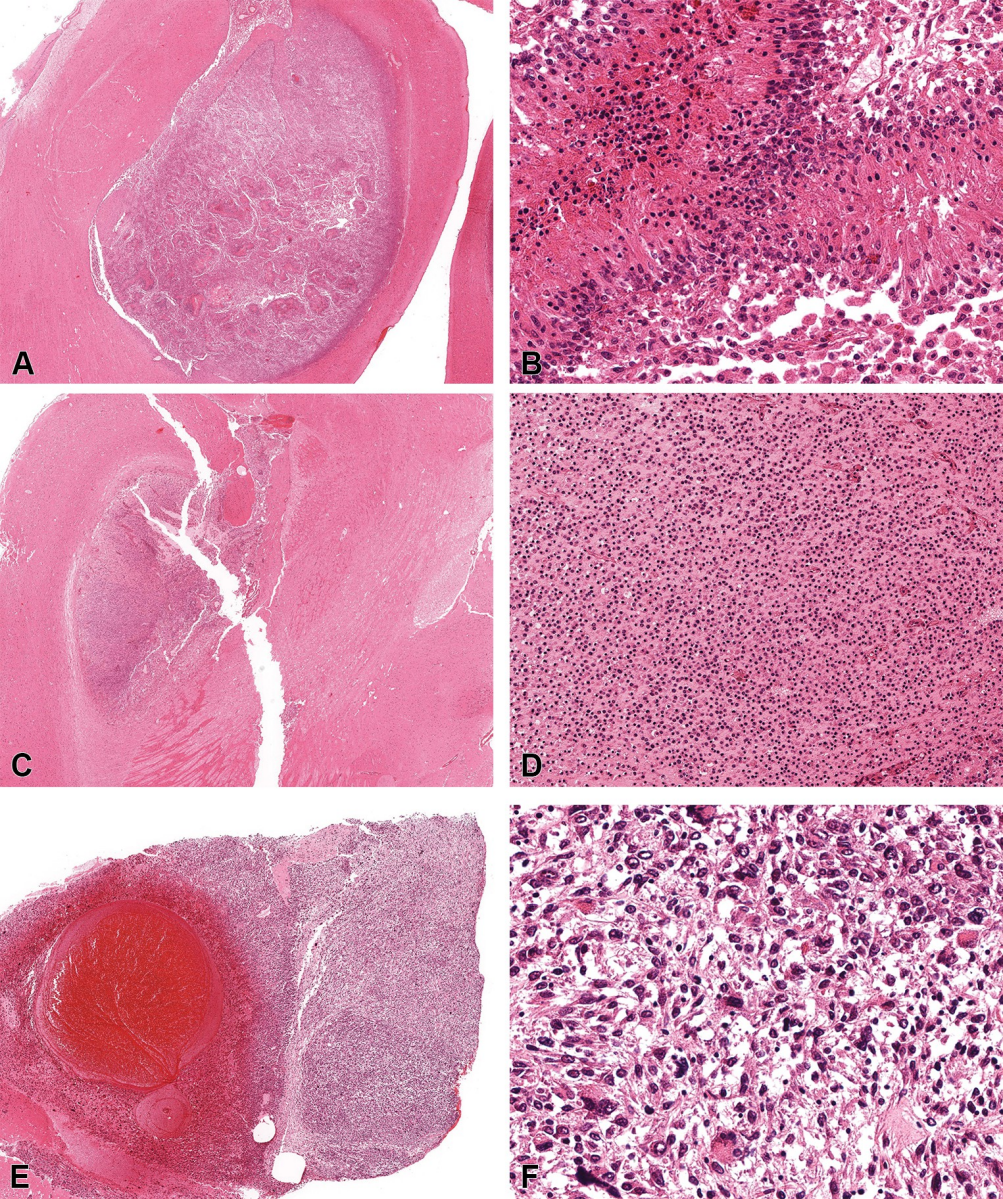
Histomicrographs of representative rat brain tumors resulting from lifetime exposure to radiofrequency radiation. All the tissues were fixed in an alcohol-based fixative and stained with hematoxylin and eosin. A-B. Astrocytoma: tumor cells with fibrillary eosinophilic cytoplasm palisading around necrotic areas. C-D. Oligodendroglioma: polygonal to round neoplastic cells with hyperchromatic nuclei. E-F. Mixed Glioma: mixed tumor cells of astrocytic and oligodendrocytic morphologies.

RFR exposure related increased incidences of malignant gliomas in the brain and schwannomas in the heart were observed in rats [12]. The malignant gliomas represented tumors of glial cell origin including astrocytoma, oligodendroglioma and mixed gliomas (specific glial cell populations between 20–80%). Due to the low incidences of glial tumors in the rodent carcinogenicity studies, different types of glial tumors are usually combined into a single category of malignant glioma. However, based on morphological features, three types of brain tumors may be discerned in rats, including astrocytoma, oligodendroglioma, and mixed gliomas. In contrast to human gliomas, rat glial tumors from carcinogenicity studies are rarely graded. Also, the relatively common human glial tumor glioblastoma is not commonly described in rats. Astrocytomas were poorly demarcated, unencapsulated, densely cellular masses composed of spindloid neoplastic cells with fibrillary eosinophilic cytoplasm and hyperchromatic nuclei, which were noted to palisade around necrotic areas and multifocally infiltrated the adjacent neuropil and the meninges (Fig 2A and 2B). Oligodendrogliomas were well-circumscribed and composed of solid cellular areas of small, uniform round to polygonal neoplastic cells with centrally located round nuclei with perinuclear halos (Fig 2C and 2D). Occasionally, clusters of glomeruloid microvascular proliferation were interspersed within the neoplastic oligodendrocytes. Mixed gliomas were poorly circumscribed brain tumors with neoplastic cells with features of astrocytes and oligodendrocytes in variable (20–80%) proportions (Fig 2E and 2F). Most of the rat gliomas in this study, graded based on the WHO human glioma criteria, were classified as low grade.

**Fig 3:**
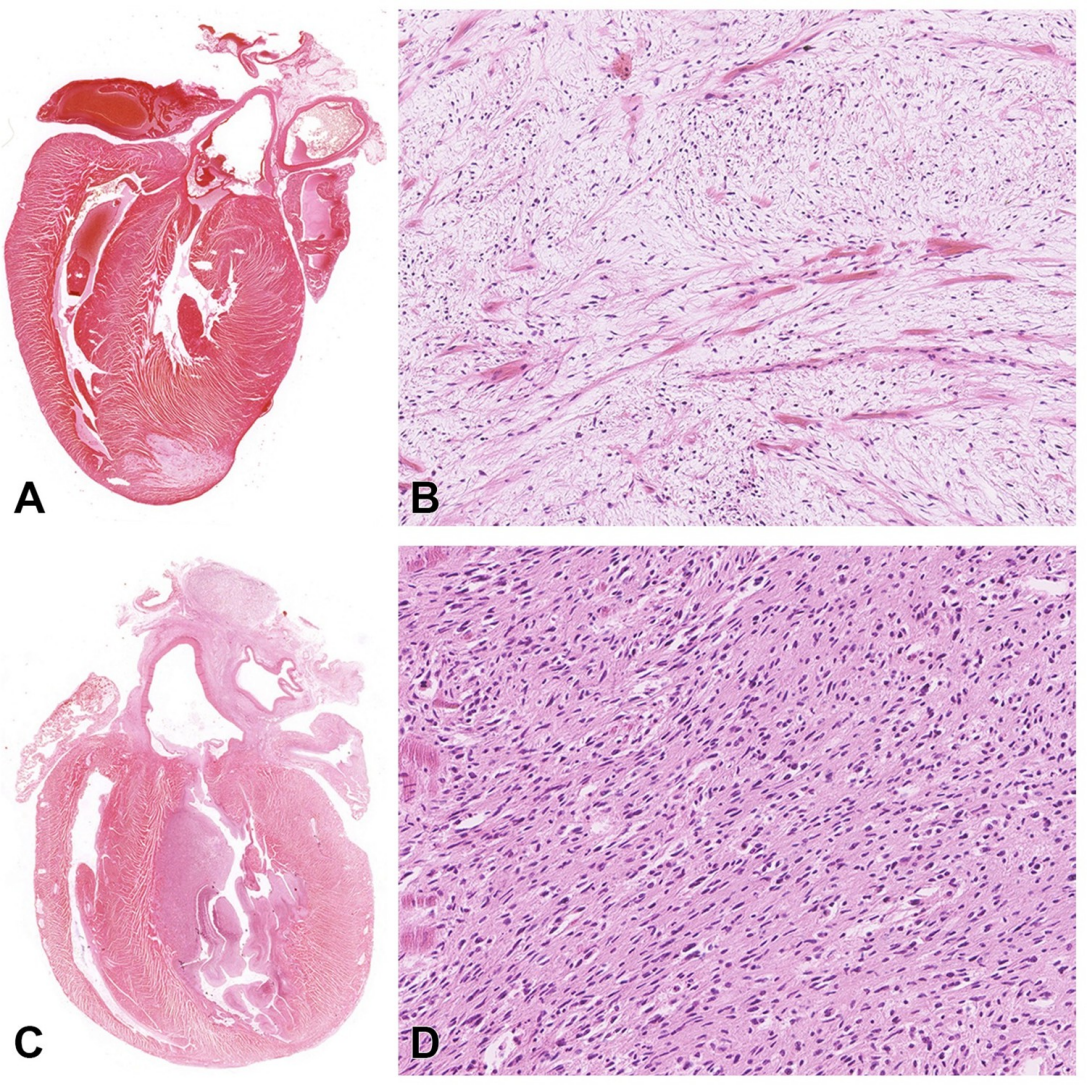
Histomicrographs of representative rat cardiac schwannomas resulting from lifetime exposure to radiofrequency radiation. All the tissues were fixed in an alcohol-based fixative and stained with hematoxylin and eosin. A-B. Intramural schwannoma involving the left ventricular wall and interventricular septum. C-D. Endocardial schwannoma.

Based on the location of the heart tumors, rat cardiac schwannomas were classified as intramural schwannoma or endocardial schwannoma. Intramural schwannomas were comprised of a neoplastic proliferation of spindle cells within the myocardium that invaded adjacent muscular layers (Fig 3A and 3B), whereas endocardial schwannomas consisted of proliferating subendocardial spindle cells that often invaded the myocardium and protruded into the ventricular lumen (Fig 3C and 3D).

### Next generation sequencing panel design and data analysis

We developed an amplicon based next generation sequencing panel of cancer genes relevant to human gliomas that would allow detection of commonly occurring orthologous mutations in rat gliomas and cardiac schwannomas. To this end, we selected 23 genes that harbor most common genetic alterations in human gliomas based on the COSMIC database, whole genome sequencing and whole exome sequencing data from human gliomas [22]. The target region spanned a total 64,759 bp across the 23 genes. An exemplary IGV plot displays coverage across the *Setd2* gene for one brain tumor (S1 Fig). All target genes were sequenced to at least 1000x coverage in brain, ENT and heart samples (S2 Fig) with the exception of *Hras* that had a mean coverage of ~100x. Amplification was not successful for some exons due to probe design issues (Table 1).

### Analysis of brain tumors

Mutation analysis was performed on 14 rat brain tumors and 9 unexposed age matched non-tumor brain controls. Twelve tumors were derived from the RFR exposed groups and 2 tumors developed spontaneously in the unexposed control group. A total of 1,111 point mutations were detected across all brain tumors of which 1,058 were exonic, 4 were within the *Tert* promoter and 49 were in either the 3’ or 5’ UTR of full-length target genes (S2 Table). The number of SNVs or indels in each rat glioma ranged from 16–234, and include synonymous, nonsynonymous (NS) and regulatory sequence alterations within the targeted genes. *Setd2* and *Pik3ca* mutations were identified in all tumors while mutations in *Nf1*, *Egfr*, *Atrx*, *Cic*, *Rb1*, *Pdgfra*, *Arid1a*, *Notch1*, *Tp53*, *Pik3r1*, *Check2*, *Idh2*, *Pten*, *Fubp1*, and *Cdkn2a* were present in at least 7/14 (50%) tumors. The remaining genes *Erbb2*, *Kras* and *Tert*, and *Braf* and *Idh1*, contained variants in five and two gliomas, respectively.

**Fig 4:**
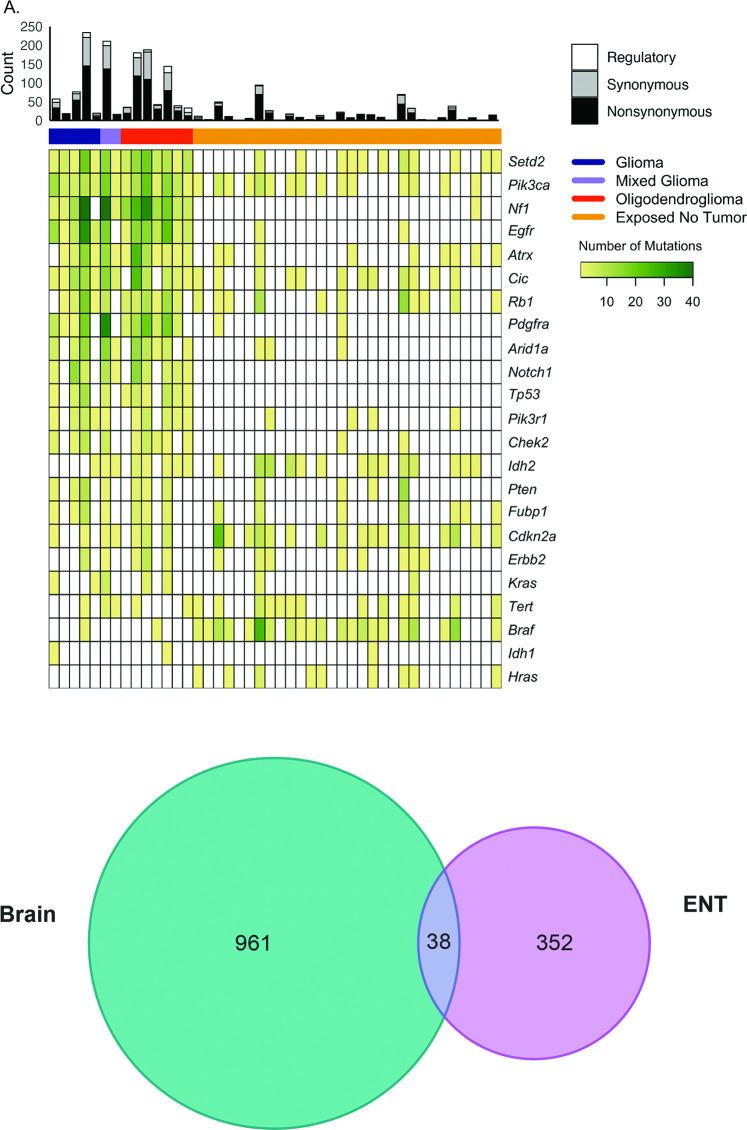
Mutation landscape of rat brain tumors and exposed non-tumor (ENT) brain samples resulting from lifetime exposure to radiofrequency radiation profiled using the targeted NGS panel. A. Venn diagram displaying count of variants unique to or shared between brain tumors and exposed non tumor (ENT) tissues. B.

Ensembl’s Variant Effect Predictor (VEP) was used to annotate SNVs and indels. Variants were first classified as ‘Nonsynonymous’, ‘Synonymous or ‘Regulatory’ (Fig 4A). For this study, the ‘Regulatory’ category included variants found in either the *Tert* promoter or 3’ UTR and 5’ UTR for genes in which all exons were targeted (Table 1). Most mutations were predicted to be NS while less than half were classified as either synonymous or regulatory. The relatively low fraction of regulatory classification was expected since these regions comprise a small portion of the total target area in the panel design.

**Fig 5:**
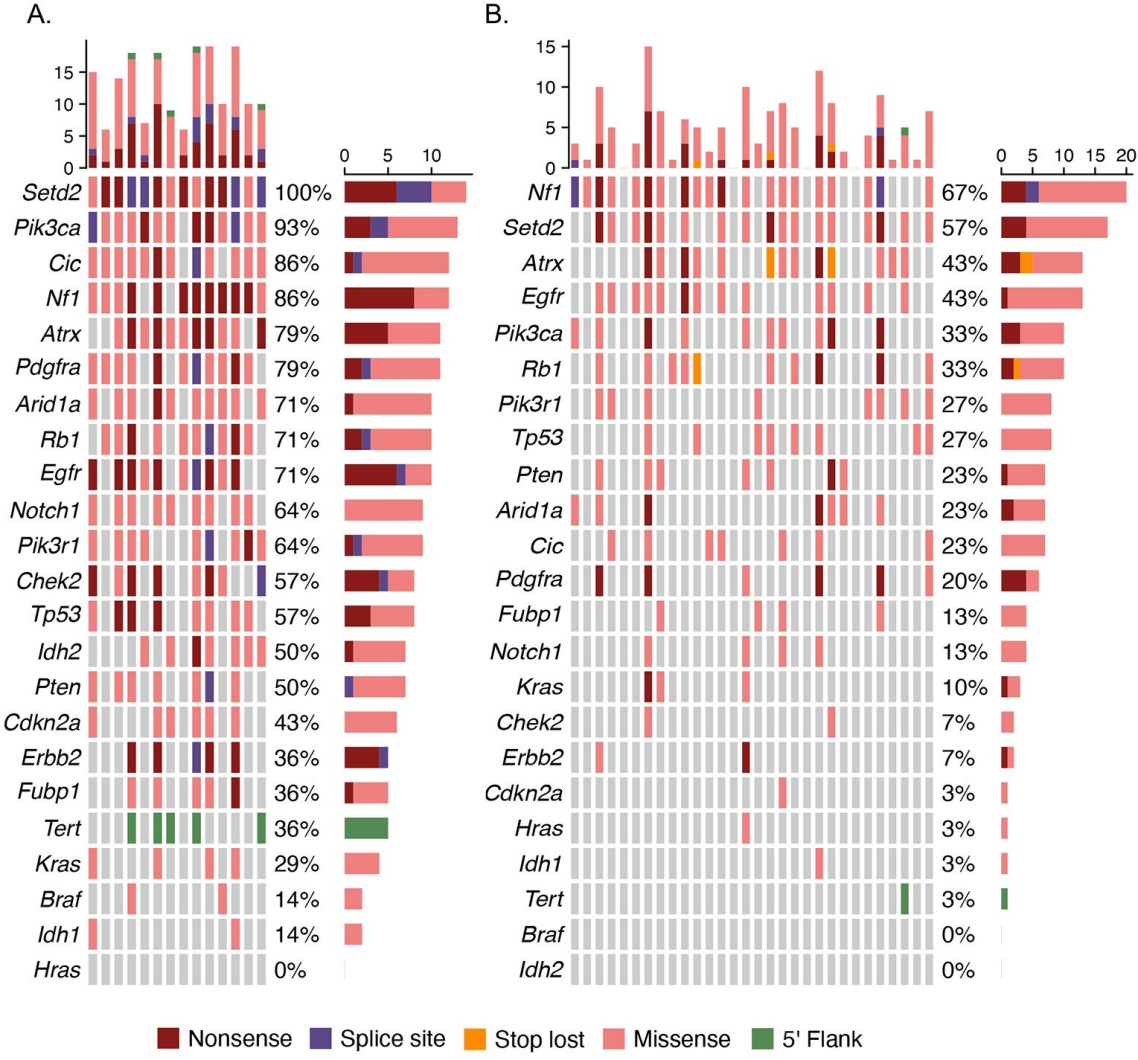
Mutation landscape of rat brain tumors (A) and exposed non-tumor (ENT) brain samples (B) resulting from lifetime exposure to radiofrequency radiation. The histograms display the distribution of most deleterious mutation types across samples (upper) and genes (right sidebar).

Nonsynonymous mutations were further classified according to their predicted functional consequence (S2 Table). Missense variants comprised the majority mutations (633), followed by nonsense (66) and splice region variants (16). Fig 5A displays the most deleterious NS mutations identified in each target gene in brain tumors. Co-occuring NS mutations were ranked with decreasing predicted deleteriousness as follows: Nonsense, Splice site, Missense. The *Tert* promoter mutations were also included in Fig 5A, although deleteriousness is not predicted in non-coding regions. The predominant deleterious mutations in *Notch1*, *Cdkn2a*, *Kras*, *Braf*, *Idh1 Setd2*, *Nf1*, *Egfr*, *Chek2* and *Erbb2* were nonsense, while missense mutations predominated in *Pik3ca*, *Cic*, *Atrx*, *Pdgfra*, *Arid1a*, *Rb1*, *Pik3r1*, *Tp53*, *Idh2*, *Pten* and *Fubp1*. Splice site mutations were the most deleterious type in *Setd2*, *Pik3ca*, *Cic*, *Pdgfra*, *Rb1*, *Egfr*, *Pik3r1*, *Chek2*, *Pten* and *Erbb2* in some tumors.

**Fig 6:**
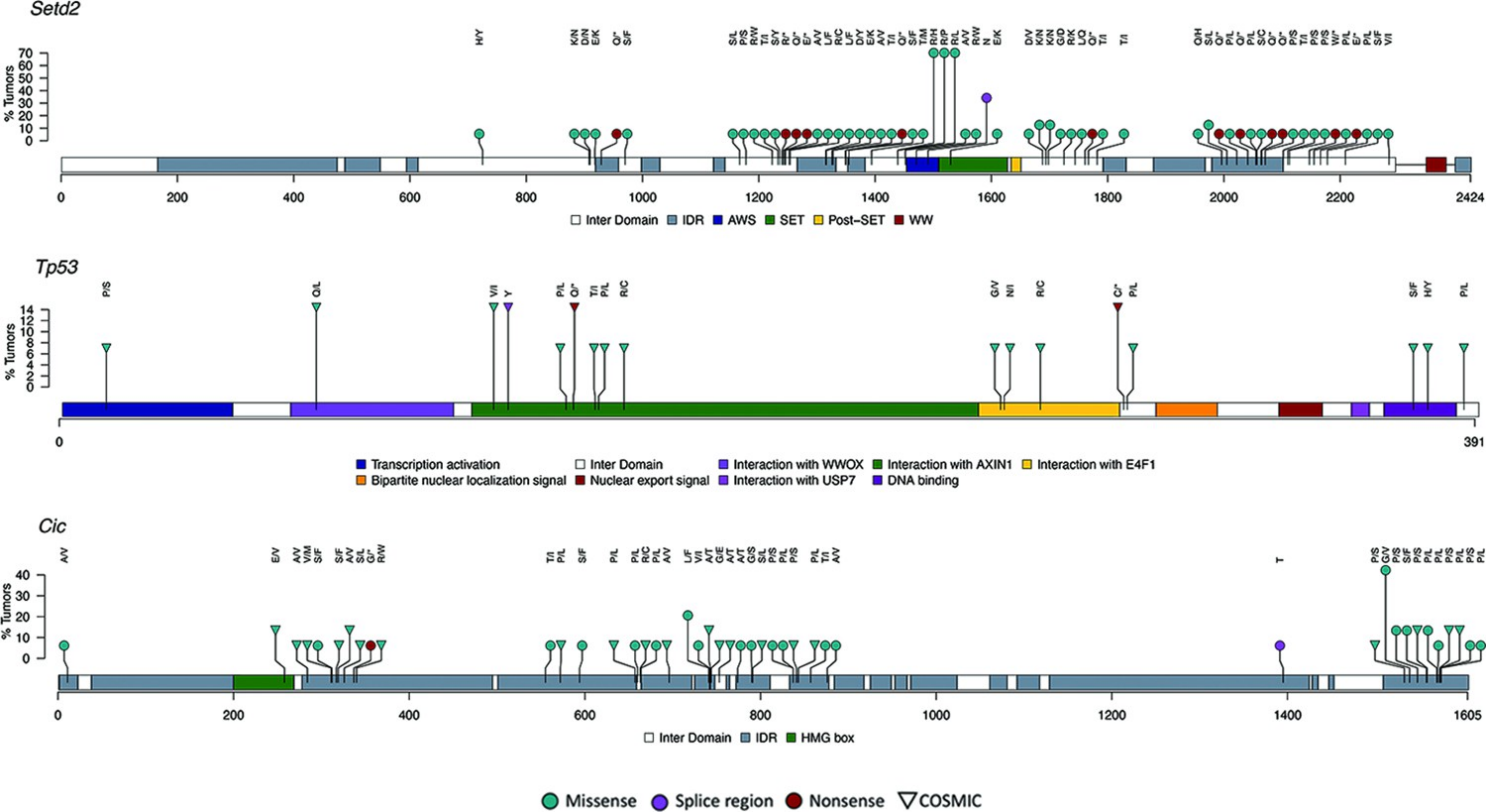
Lolliplots show distribution of non-synonymous SNVs in selected cancer related genes in rat brain tumors resulting from lifetime exposure to radiofrequency radiation. Mutations are color-coded according to predicted functional consequence and relevance to known orthologous human mutations are indicated with an upside-down triangle.

The most frequently altered residue in the brain tumors was *Setd2* p.1439R (Fig 6, S2 Table). Three separate missense mutations, p.1439R>P/L/H, were detected at this codon in 10/14 tumors classified as either exposed glioma, mixed glioma, oligodendroglioma or unexposed spontaneous oligodendroglioma. A missense mutation at *Cic* p.1539G>V occurred in 6/14 tumors classified as either exposed glioma, unexposed spontaneous glioma, mixed glioma or oligodendroglioma. The remaining mutations occurred in <6 tumors with the large majority (854/999) occurring in one tumor (S2 Table). Two missense mutations were detected in the *Idh1*, each occurring once in separate tumors. Eight separate NS mutations occurred in *Idh2*, with six of those (p.178Q, p.174A, p.150E, p.147V, p.138T, p.130K) occurring each once in single separate tumor and two mutations (p.176G, p.177D) each occurring in more than one tumor.

No mutations were detected in rat gliomas that had homology to the human *IDH1* p.132R or *IDH2* p.172R mutations suggesting that rat gliomas are primarily wild-type for IDH hotspot mutations implicated in human gliomas (S2 Table). Furthermore, the *Idh*1 and *Idh2* mutations identified in rat gliomas did not have homology to known human variants in COSMIC database. Four *Tert* promoter SNVs were detected in the rat gliomas, three of which occurred once in separate tumors. One SNV at position 32,273,028 in the promoter was detected in four tumors including ‘Glioma 4’, ‘Mixed glioma 2’, ‘Oligodendroglioma 2’ and the spontaneous oligodendroglioma indicating that this mutation could be important in glioma tumorigenesis in rat.

### Analysis of non-tumor interim exposure group

We hypothesized that detectable early mutation events occur in non-tumor brain tissue subjected to short term RFR exposure. To explore this hypothesis, variant analysis was performed on non-tumor brain tissue from rats exposed to RFR for one year and subsequently compared to SNVs from brain tumors collected after lifetime exposure. In brief, brain specimens were examined from rats exposed to 50 V/m (high dosage, ‘HD’), 25 V/m (mid dosage, ‘MD’) or 5 V/m (low dosage, ‘LD’) from the interim period with no incurring tumor development (e.g. Exposed Non-tumor Tissues (ENTs)). Ten specimens per dosage group and 10 unexposed normal controls were analyzed as previously described. Overall, there were fewer variants identified in the ENTs (432 total unique, average = 14 variants/ENT) relative to the brain tumors (1,111 total unique, average = 79 variants/tumor) (S3 Table). *Nf1*, *Setd2* and *Egfr* contained variants in at least 17/30 (50%) of all ENTs (Fig 4A). Mutations were detected in *Atrx*, *Rb1*, *Pik3ca*, *Arid1a* and *Tp53* in at least 13/30 (50%) of ENTs. *Cic*, *Pik3r1*, *Pten*, *Pdgfra*, *Notch1*, *Fubp1*, *Kras*, *Chek2*, *I dh1*, *Erbb2*, *Hras*, *Cdkn2a* and the *Tert* promoter contained variants in < 30% of samples and no mutations were detected in *Braf* or *Idh2*. The average number of variants per ENT for the HD, MD and LD groups was 17.4, 9.3 and 21.5, respectively, suggesting that RFR dosage level may not be associated with frequency of mutation events in the target genes.

There were 397 distinct changes within 390 unique residues. Similar to the brain tumors, the majority of changes were nonsynonymous (298/397) (S3 Table). The most frequent alteration was a missense mutation at *Nf1* p.1772I>S that occurred in 7/30 ENTs. It is interesting to note that the most common mutation in brain tumors, *Setd2* R1439, was mutated in 4/30 ENTs. Thirty eight mutations were shared between brain tumors and ENTs (Fig 4B). A single *Idh1* NS mutation without COSMIC relevance was found in one ENT and no alterations were detected in *Idh2* (S3 Table).

### Analysis of rat cardiac schwannomas

**Fig 7:**
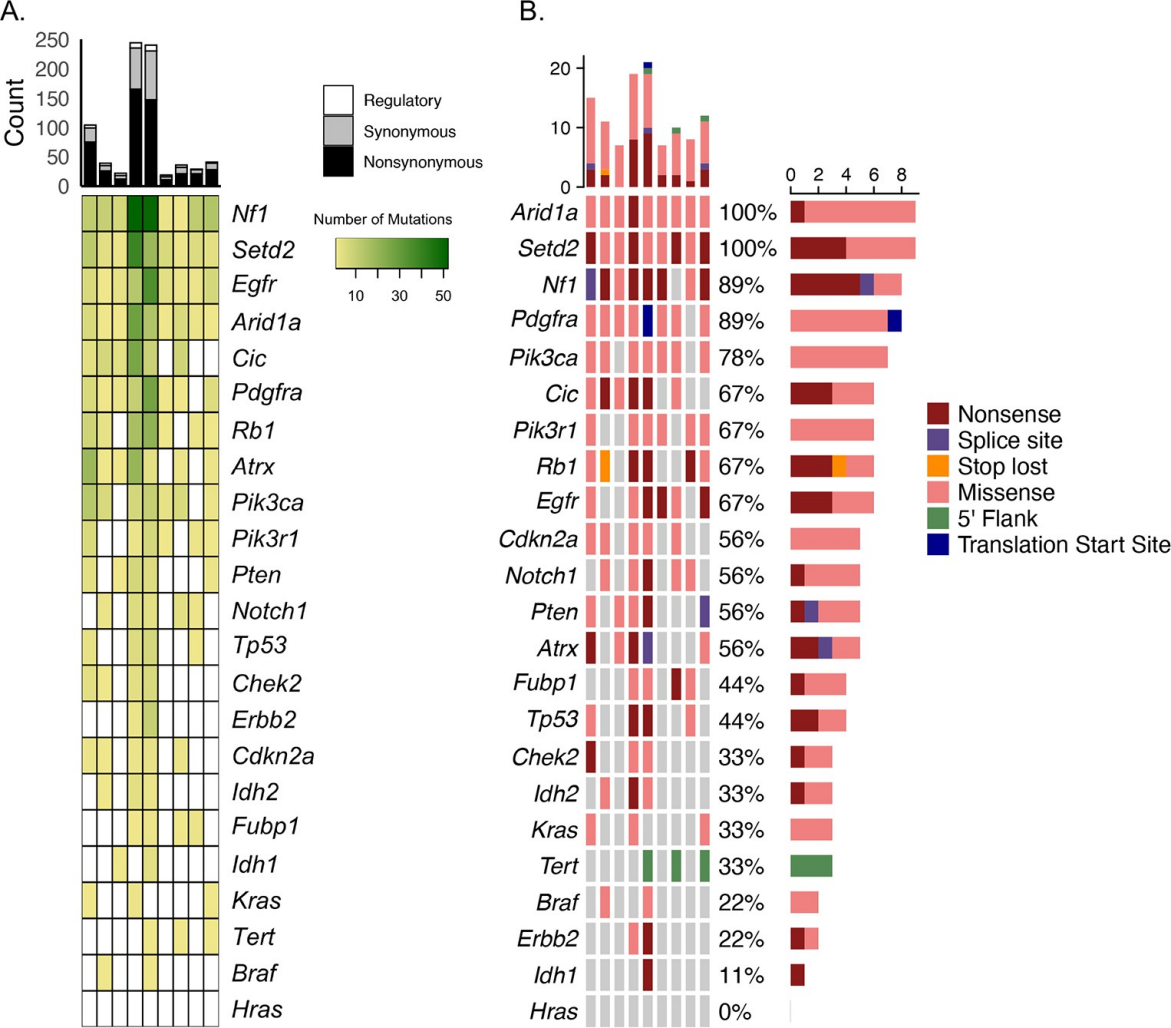
Mutation landscape of rat cardiac schwannomas resulting from lifetime exposure to radiofrequency radiation. The heatmap displays frequency of all mutations identified in tumors. A. The mutation landscape of most deleterious mutation identified in each gene for cardiac tumors. B.

**Fig 8:**
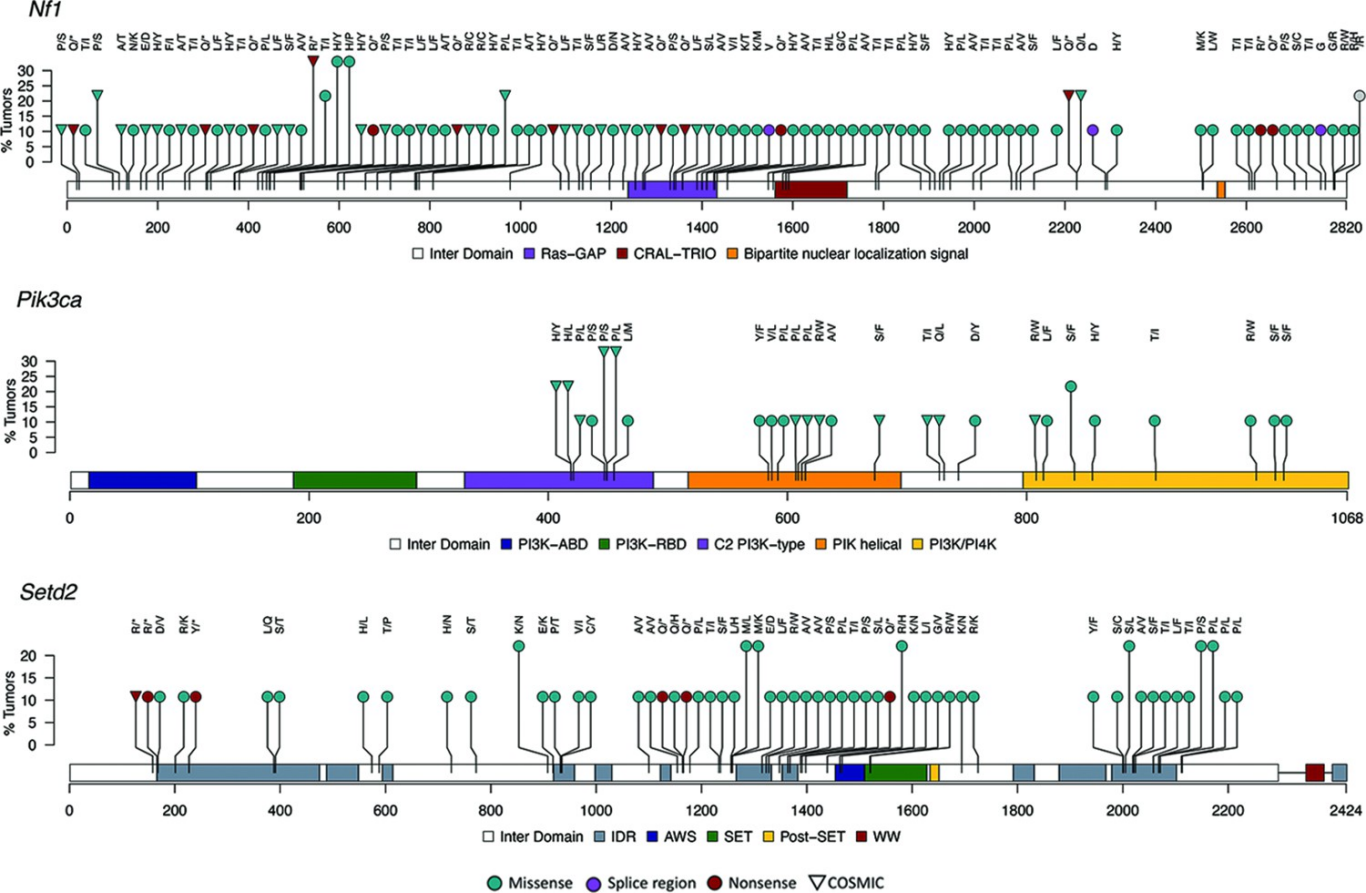
Lolliplots show distribution of non-synonymous rat SNVs in selected cancer related genes in rat cardiac schwannomas resulting from lifetime exposure to radiofrequency radiation. Mutations are color-coded according to predicted functional consequence and relevance to known orthologous human mutations is indicated with an upside-down triangle.

Mutation analysis was performed on 9 cardiac schwannomas and 9 unexposed age-matched non-tumor hearts from controls. Eight tumors were obtained from rats subjected to lifetime RFR exposure and one tumor developed spontaneously in an unexposed control. A total of 744 point mutations were detected in exons (716), the *Tert* promoter (1) and 3’UTR or 5’UTR (27) across all tumors (S4 Table). Mutations were found in *Arid1a*, *Setd2*, *Nf1* and *Egfr* in all cardiac tumors and in *Pdgfra* for eight of the tumors (Fig 7A). *Rb1*, *Atrx*, *Pik3ca*, *Cic*, *Pik3r1*, *Pten*, *Notch1* and *Cdkn2a* contained mutations in 5–7 of the tumors while *Tp53*, *Chek2*, *Fubp1*, *Idh2*, *Kras*, *Tert*, *Erbb2*, *Idh2* and *Braf* mutations were identified in less than half of the tumors. Similar to glioma results, NS mutations occurred more frequently than those classified as synonymous or regulatory. Missense mutations were the most common deleterious NS type detected in *Arid1a*, *Setd2*, *Pdgfra*, *Pik3ca*, *Pik3r1*, *Cdkn2a*, *Notch1*, *Pten*, *Fubp1*, *Kras and Braf* (Fig 7B). The frequency of the most deleterious missense and nonsense mutations were proportional in *Cic*, *Egfr*, *Atrx*, *Tp53* and *Erbb2* in the tumors. For 3 tumors, the most deleterious mutation was predicted to impact a splice site in *Nf1*, *Pten* and *Atrx*. A stop loss mutation in *Rb1* and a translation start site mutation in *Pdgfra* were the most deleterious type detected in two separate cardiac tumors. A total of 701 distinct changes occurred in 688 residues. The most frequent (3/9 tumors) alterations occurred at *Nf1* p.440R, *Nf1* p.448H and *Pik3ca* p.449P (Fig 8). A stop gain was acquired for *Nf1* p.440R while *Nf1* p.448H and *Pik3ca* p.449P acquired missense mutations. The most frequent alteration in brain tumors, *Setd2* R1439, was also found in 2/9 heart tumors.

### Translational relevance of rat mutations to human cancers

We queried the COSMIC database to understand the translation relevance of mutations in rat glioma and cardiac schwannomas to mutations in orthologous genes in human cancers. Greater than 50% of variants in *Cdkn2a*, *Erbb2*, *Pik3r1* and *Tp53* and less than 50% in *Arid1a*, *Braf*, *Egfr*, *Nf1*, *Pdgfra* and *Pik3ca* were described in COSMIC for both brain and heart tumors (S2 and S4 Tables). While *Setd2* variant in one cardiac schwannoma was described in COSMIC, none of the *Setd2* variants in gliomas were in the database. On the other hand, there were notably more *Kras* mutations in gliomas (10/12, 83%) that were in COSMIC compared to *Kras* mutations in cardiac schwannomas (2/4, 50%). All 13 *Tp53* NS mutations in gliomas had orthologous alterations annotated in COSMIC and eight (61%) of those were associated with central nervous system tissue (CNS) tumors (Fig 6). Somewhat similarly in cardiac schwannomas, 12/15 mutations were orthologous to human mutations in COSMIC and three (25%) of those were associated with CNS tissue.

Of the 1,111 point mutations in rat gliomas, 282 had homology to mutations in COSMIC and 187 of those were predicted to be NS in rat (S2 Table). Of the 187 NS orthologous mutations, 28 were implicated in human gliomas in COSMIC. Most rat mutations with homology to mutations implicated in human gliomas occurred in one tumor. However, mutations at *Cic* p.319A. *Tp53* [p.71Q, p.124Y, p.142Q] and *Nf1* [p.440R, p.1080S] were found in at least 2/14 tumors (Fig 6). Of the 28 NS orthologous mutations implicated in human gliomas, 24 were classified as pathogenic in COSMIC and 17 have conserved amino acid change for rat and human including *Tp53* [p.140P>L, p.142Q>*, p.149P>L, p.156R>C, p.260G>V, p.271R>C], Nf1 [p.192R>*, p.379C>Y, p.436S>L, p.440R>*, p.758Q>*, p.1080S>L], *Erbb2* p.39P>S, *Egfr* p.596P>S, *Pik3r1* p.464D>N, *Cdkn2a* p.26A>T and *Arid1a* p.>E.

Of the 744 cardiac schwannoma point mutations, 205 were orthologous to COSMIC mutations and 146 of those were classified as NS (S4 Table). The orthologous NS mutations were found in *Cic*, *Tp53*, *Nf1*, *Erbb2*, *Chek2*, *Egfr*, *Pdgfra*, *Pik3ca*, *Fubp1*, *Pik3r1*, *Kras*, *Braf*, *Cdkn2a*, *Arid1a*, *Setd2 and Idh1*. Taken together, these results suggest that rat glioma and cardiac schwannoma mutation profiles have 25–27% similarity to known human mutations but most target genes investigated in the panel harbor alterations that are distinct to rat. Genes that appear to be an exception to this are *Cdkn2a*, *Erbb2*, *Pik3r1 and Tp53* as well as *Fubp*1 in the heart tumors.

## Discussion

In this study we have demonstrated that the gliomas and cardiac schwannomas in rats resulting from lifetime exposure to low dose far field RFR that are used for cellular telephone communications, are morphologically similar to low grade human gliomas and that about 25% of the mutations seen in these tumors have corresponding alterations in homologs of human cancer genes. Surprisingly, none of the rat gliomas examined in this study harbored mutations in *Idh1/2* genes that are common in human gliomas. We have accomplished this by developing the first targeted glioma NGS gene panel for rats to study the molecular changes in rat gliomas. Gliomas and schwannomas are rare in the rat and there are no reports of genetic alterations in these tumors in rat, which is an important model organism in chemical carcinogenesis studies. Gliomas and cardiac schwannomas derived from life-time RFR exposure studies performed at the RI provided a unique opportunity to fill this gap in knowledge. In addition to tumors, we were able to examine RFR interim exposed non-tumor brain tissue (ENTs) for early molecular changes that could contribute to tumorigenesis. Twenty three cancer-related genes were selected based on their relevance to human gliomas, tumors of the central nervous system or those with broad relevance to several cancer types.

### Relevance of mutations in rat gliomas and cardiac schwannomas compared to human cancers

We discovered that rat tumors lack the *Idh1* R132 and *Idh2* R172 hotspot mutations that are widely implicated in human gliomas (mainly in WHO grade II and III gliomas and secondary glioblastoma). These results are comparable to our preliminary results based on Sanger sequencing of a distinct set of rat gliomas that originated spontaneously or due to chronic chemical exposures (*data not presented*). Interestingly, the absence or rarity of mutations in *IDH1* R132 and *IDH2* R172 was also demonstrated in gliomas from dogs, another important model system with significant human relevance [22, 23]. *Idh1/2* did harbor other nonsynonymous mutations but only three of their human homologs were found in the COSMIC database and the functional significance of those alterations remains to be tested. This was an unexpected discovery given the prevalence of IDH mutations in human gliomas. Compared to gliomas with wild-type IDH, gliomas with IDH1/2 mutations have better prognosis and outcomes, and these genes have become potential therapeutic targets for gliomas [24, 25]. While tremendous progress has been made towards identifying important IDH mutation profiles, co-occurrence with other genetic alterations can be more predictive for tumor classification and prognosis [24, 26]. For example, prognosis can be stratified based on co-occurrence of IDH mutations with presence or absence of 1p/19q deletion, as well as mutations in the *Tert* promoter, *Tp5*3 and *Egfr*. Despite the absence of *Idh1/2* mutations in rat and canine gliomas, these model organisms with wild-type IDH gliomas harbor mutations in other genes relevant for human glioma research.

In addition to *IDH*, mutations in the *Tert* promoter contribute to glioma classification and may have prognostic clinical value [26]. Furthermore, 70–74% of glioblastomas and oligodendrogliomas contain mutations in the *Tert* promoter [27]. TERT is the catalytic subunit of telomerase and mutations in this gene lead to telomerase activation in gliomas. We detected remarkably few mutations in the *Tert* promoter. An interesting discovery was that the *Tert* g.32,273,028C>T SNV was found in 4/14 gliomas and 3/9 cardiac schwannomas. This mutation is within a region of poor homology to human gene and thus the potential relevance to human cancer is not known at this time. However, this SNV is a candidate for follow up evaluation given its prevalence in the RFR exposed rat tumors.

In humans, two hotspot *TERT* promoter mutations, C228T and C250T (Hg19), are associated with several cancers. Recently, these mutations were confirmed in tumors as well as in circulating cell-free DNA from glioma patients [28] and are seen in majority (~75%) of IDH wild-type glioblastomas. We did not detect these alterations in the rat gliomas, ENTs or schwannomas. Taken together, these results suggest that there are distinct differences in the relevance of *Idh* and *Tert* promoter mutations between rat and human tumors. More clear patterns of mutation association could emerge if the study is extended to include detection of the 1p/19q deletion, for example, performing whole exome or whole genome sequencing if more samples are available for examination. Furthermore, it’s possible genomic alterations incurred due to RFR exposure could be distinct from those examined in the majority of human studies because of the nature of the mutagenic processes leading to tumor development and progression.

Similar to *Idh1/2*, mutations in *Atrx*, *Notch1*, *Pten*, *Rb1* and *Setd2* were largely absent from COSMIC. This was particularly surprising for *Setd*2 given that it was altered in every glioma and cardiac schwannoma in this study, with only one nonsense mutation (*SETD2* p158R> *) reported in the COSMIC database. It is notable that this alteration is associated with human tumors of the central nervous system. In humans, *SETD2* is responsible for methylation of H3K36 and loss of function (LOF) leads to decreased H3K36me3 levels. Reduced H3K36me3 levels disrupt gene regulation and chromatin stability and is associated with tumor development [29]. In humans, an association was determined between high-grade gliomas and *SETD2* LOF, specifically due to alterations that result in protein truncation [30]. In the rat tumors, 20%, 11% and 11% of NS mutations were predicted to be nonsense in the gliomas, ENT brains and cardiac schwannomas, respectively. It is possible that *Setd2* LOF, irrespective of precise mutation position, contributes to tumorigenesis in rat as indicated in humans. The *Setd2* R1439 residue was one of the most frequently altered amino acids with missense mutations detected in 10/14 gliomas, 4/30 ENT brains and 2/9 cardiac schwannomas. Relevance of this mutation to human cancer is not described in COSMIC or reported in the scientific literature at this time. Investigation revealed the residue lies with a region of *Setd2* that is involved in an interaction with α-tubulin and disruption of tubulin methylation by *Setd2* contributes to genomic instability [31]. Further examination and validation of this variant should be considered.

In contrast to the above genes with mutations that seemingly have low relevance to human cancers, alterations in *Tp53*, *Cdkn2a*, *Erbb2*, *Chek2*, *Kras* and *Pik3r1* with homology to COSMIC variants were relatively common in rat gliomas and cardiac schwannomas (e.g. > = 50%). All 13 *Tp53* NS mutations in gliomas had orthologous alterations annotated in COSMIC and 61% of those were associated with CNS tumors; somewhat similarly, most *Tp53* mutations (12/15) in cardiac schwannomas were orthologous to mutations in human genes in COSMIC. *Pik3ca*, which was altered in the majority of tumors, harbored NS mutations in gliomas (40%) and cardiac schwannomas (46%) with COSMIC relevance, however in contrast to *Tp53*, they are not associated with gliomas or CNS tumors at this time.

Less than half of the mutations in *Pdfra*, *Fubp1*, *Nf1*, *Egfr*, *Braf*, *Pik3ca*, *Cic* and *Arid1a* were described in COSMIC suggesting that there are some mutations in these genes that are unique to rat or are unknown in human at this time. *Pik3ca* NS mutations were detected in 13/14 (93%) brain tumors and 7/9 (78%) heart tumors. Of the 52 NS mutations in rat gliomas, 21 (40%) are described in the COSMIC, yet none are implicated in human gliomas or other tumors of the central nervous system. Similarly in cardiac schwannomas, 12/26 (46%) *Pik3ca* NS variants were described in COSMIC without relevance to central nervous system tumors. Interestingly, we detected a c.3040C>T nonsense mutation in 3/14 gliomas which is not in the COSMIC database but was reported as a human nonsense mutation [32]. These results suggest that *Pik3ca* mutations might be associated with rat glioma and schwannoma tumor progression but the relationship to tumor type seem to differ between human and rat. It is interesting to note that *Arid1*a NS mutations were detected in all cardiac schwannomas and 41% of these were orthologous to COSMIC variants associated with a variety of human cancers. Meanwhile, 10/14 of gliomas harbored NS mutations in *Arid1a* and 43% of those were orthologous to COSMIC variants. An exome study in humans found that 37/125 (29%) of schwannomas have NS mutations in *ARID1A* or *ARID1B* [33]. *ARID1A* and *ARID1B* are components of the SWI-SNF complex that are involved in chromatin remodeling and are implicated in several human cancers. It is possible that the significance of *Arid1a* to schwannoma progression may be similar in the rat.

### Limitations

Despite lifetime RFR exposure and a large sample size of rats in each exposure group, tumor incidences in our study were low which limited the power of our study design. It is difficult to perform rigorous statistical testing or draw strong conclusions about mutational patterns for a tumor population based on a limited sample size. In general, identification of false positives in somatic variant detection is complicated by the occurrence of low frequency variants that approach known sequencing error rates. Recent advancements in sequencing strategies, such as error corrected duplex sequencing and NanoSeq, have potential to improve the precision of variant identification through reduced error rates in base calling [34, 35]. Further, tumor heterogeneity may contribute to variability in VAF both within tumor and across tumors from different rats. Typically, a large sample size can improve the ability to distinguish between false positives and true variants in the population. In addition to a relatively small sample size, there are no known rat glioma or schwannoma variants described in the literature and cross-species validation is preliminary at this time.

In spite of these challenges, we were able to identify variants that are likely to be important in rat glioma and cardiac schwannoma progression and warrant further examination. The availability of genomic matched controls from kidney tissue of the RFR exposed rats enabled us to exclude germline variants. We also employed two separate variant callers including Strelka2, which is commonly used in the clinical setting, and deepSNV which is designed to detect rare variants in targeted amplicon data. Evaluation of variant quality metrics and sequencing depth revealed variability across samples such that, after much consideration, a single set of cutoffs did not seem appropriate. Thus, in contrast to most mutational studies that apply broad cutoffs to remove false positives, we applied customized, adaptable filters that we were determined separately for each sample. Variants from unexposed non-tumor brain and heart tissues were removed to further reduce false positives. Finally, only variants that were present in both Strelka2 and deepSNV filtered calls were retained to achieve high confidence variant sets.

In summary, our results demonstrate that regardless of their etiology (due to lifetime RFR exposure or arising spontaneously), rat gliomas are primarily *Idh*1/2 wild type unlike most human gliomas. Histologically, most of the rat gliomas resemble diffuse low-grade gliomas in humans and such gliomas that do not harbor *IDH1/2* mutations in humans are known to have poor prognosis. The genetic alterations in other cancer genes evaluated in this panel provide novel insights into tumor progression in rat gliomas and cardiac schwannomas. The relevance of specific mutations to human cancers is variable, with some genes (*Tp53*, *Cdkn2a*, *Erbb2*, *Chek2*, *Kras* and *Pik3r1*) harboring many alterations with COSMIC relevance while the opposite is true for other target genes (*Idh1/2*, *Atrx*, *Notch1*, *Pten*, *Rb1* and *Setd2*). Several of these conserved mutations in rat tumors do not have comparable alterations in the COSMIC database, suggesting that the orthologous mutations could have different functional consequences in rat carcinogenesis and deserve further study. An important consideration is that molecular differences underlying mutational processes contribute to distinct mutational patterns which could be the result of similar etiology, albeit by different mechanisms.

Several of the variants that were detected in gliomas were also observed in non-tumor brain tissues from interim time point providing an insight into the molecular pathogenesis in rodent carcinogenicity studies and these strategies may be utilized to potentially estimate the cancer hazard risk in shorter term animal studies. Finally, this targeted mutation panel may be refined using data from whole genome or exome sequencing of rat tumors and performing error corrected duplex sequencing to increase the sensitivity to detect rare mutations in exposed non-tumor tissues from early time points.

## Supporting information

S1 Fig Integrated Genomics Viewer (IGV) browser image displays read pile-up across targeted regions of *Setd2* in a brain tumor. (TIF)

S2 Fig Sequencing depth of all target genes is indicated. All the genes were sequenced to at least 1000x coverage in brain, ENT and heart samples with the exception of *Hras* that had a mean coverage of ~100x.
(TIF)

S1 Table Description of the HSD rat tissue samples obtained from life-time exposure to Radio Frequency Radiation (RFR) studies from the Ramazzini Institute. (XLSX)

S2 Table Details of rat brain variants including positional information, predicted changes and functional consequences. Where relevant, homologous protein position in human and COSMIC inclusion is indicated.
(XLSX)

S3 Table Details of rat ENT variants including positional information, predicted changes and functional consequences. Where relevant, homologous protein position in human and COSMIC inclusion is indicated.
(XLSX)

S4 Table Details of rat heart variants including positional information, predicted changes and functional consequences. Where relevant, homologous protein position in human and COSMIC inclusion is indicated.
(XLSX)

## Abbreviations

CNTL: Control
CNS: Central nervous system
COSMIC: Catalogue of somatic mutations in cancer
FC: Fold change
H & E: Hematoxylin and Eosin
HSD: Harlan Sprague Dawley
IDH: Isocitrate dehydrogenase
IGV: Integrative genome viewer
LOF: Loss of function
NGS: Next generation sequencing
NS: Nonsynonymous
NTP: National Toxicology Program
RFR: Radiofrequency radiation
ROC: Report on carcinogens
SNV: Single nucleotide variant
SPNT: Spontaneous
