# Correction: Genetic profiling of rat gliomas and cardiac schwannomas from life-time radiofrequency radiation exposure study using a targeted next-generation sequencing gene panel

**DOI:** 10.1371/journal.pone.0346498

**Published:** 2026-04-02

**Authors:** Ashley M. Brooks, Andrea Vornoli, Ramesh C. Kovi, Thai Vu T. Ton, Miaofei Xu, Ahmed Mashal, Eva Tibaldi, Federica Gnudi, Jian-Liang Li, Robert C. Sills, John R. Bucher, Daniele Mandrioli, Fiorella Belpoggi, Arun R. Pandiri

In the Analysis of non-tumor interim exposure group subsection of the Results, there is an error in the sixth and seventh sentences of the first paragraph. The correct sentences are: Nf1, Setd2 and Egfr contained variants in at least 15/30 (50%) of all ENTs (Fig 4A). Mutations were detected in Atrx, Rb1, Pik3ca, Arid1a and Tp53 in at least 11/30 (37%) of ENTs.

Fig 4 was uploaded incorrectly. Please see the correct Fig 4 here

**Fig 4:**
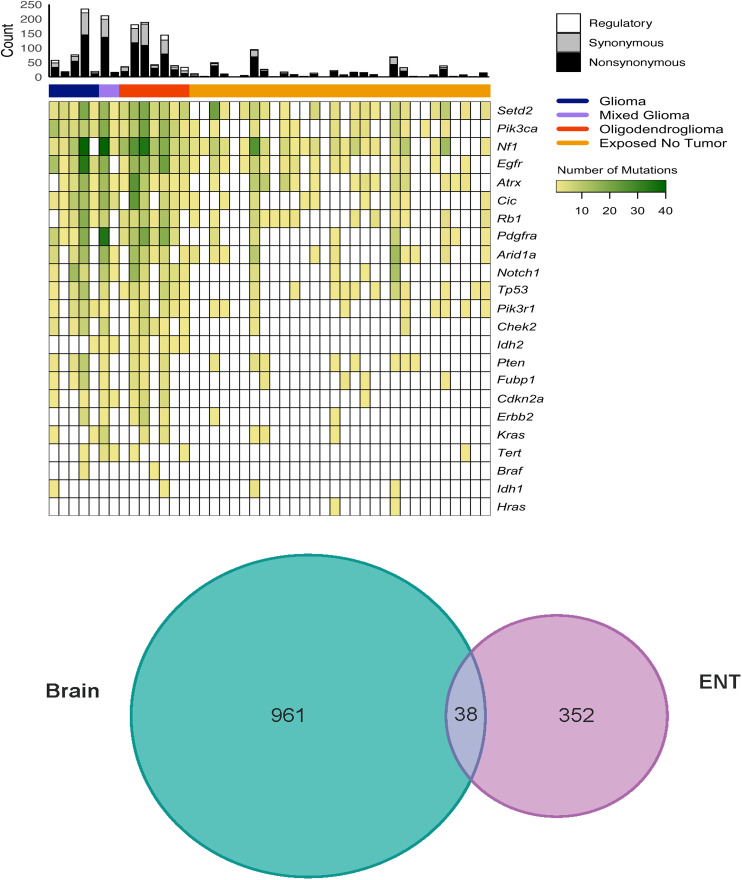
Mutation landscape of rat brain tumors and exposed non-tumor (ENT) brain samples resulting from lifetime exposure to radiofrequency radiation profiled using the targeted NGS panel. A. Venn diagram displaying count of variants unique to or shared between brain tumors and exposed non tumor (ENT) tissues. B.
