# Supplementary figures and images for: Genetic profiling of rat gliomas and cardiac schwannomas from life-time radiofrequency radiation exposure study using a targeted next-generation sequencing gene panel
Source: PLoS One. 2024 Jan 17;19(1):e0296699. doi: 10.1371/journal.pone.0296699 (PMC10793937; doi:10.1371/journal.pone.0296699)

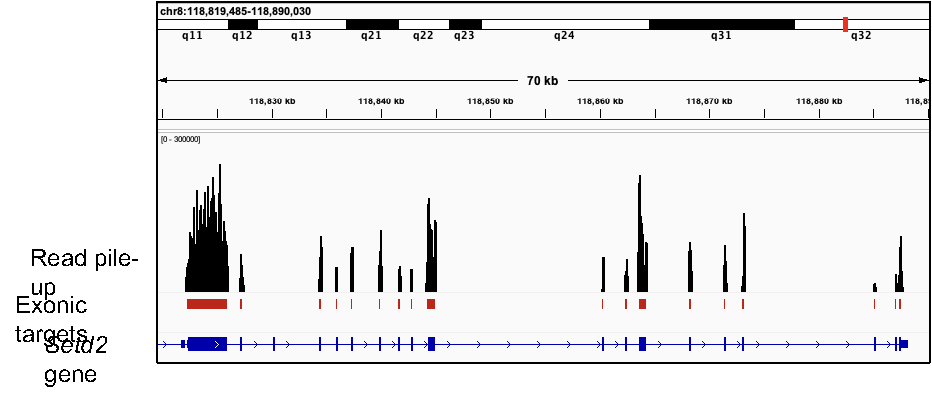

Supplement: S1 Fig — (TIF) [file pone.0296699.s001.tif]

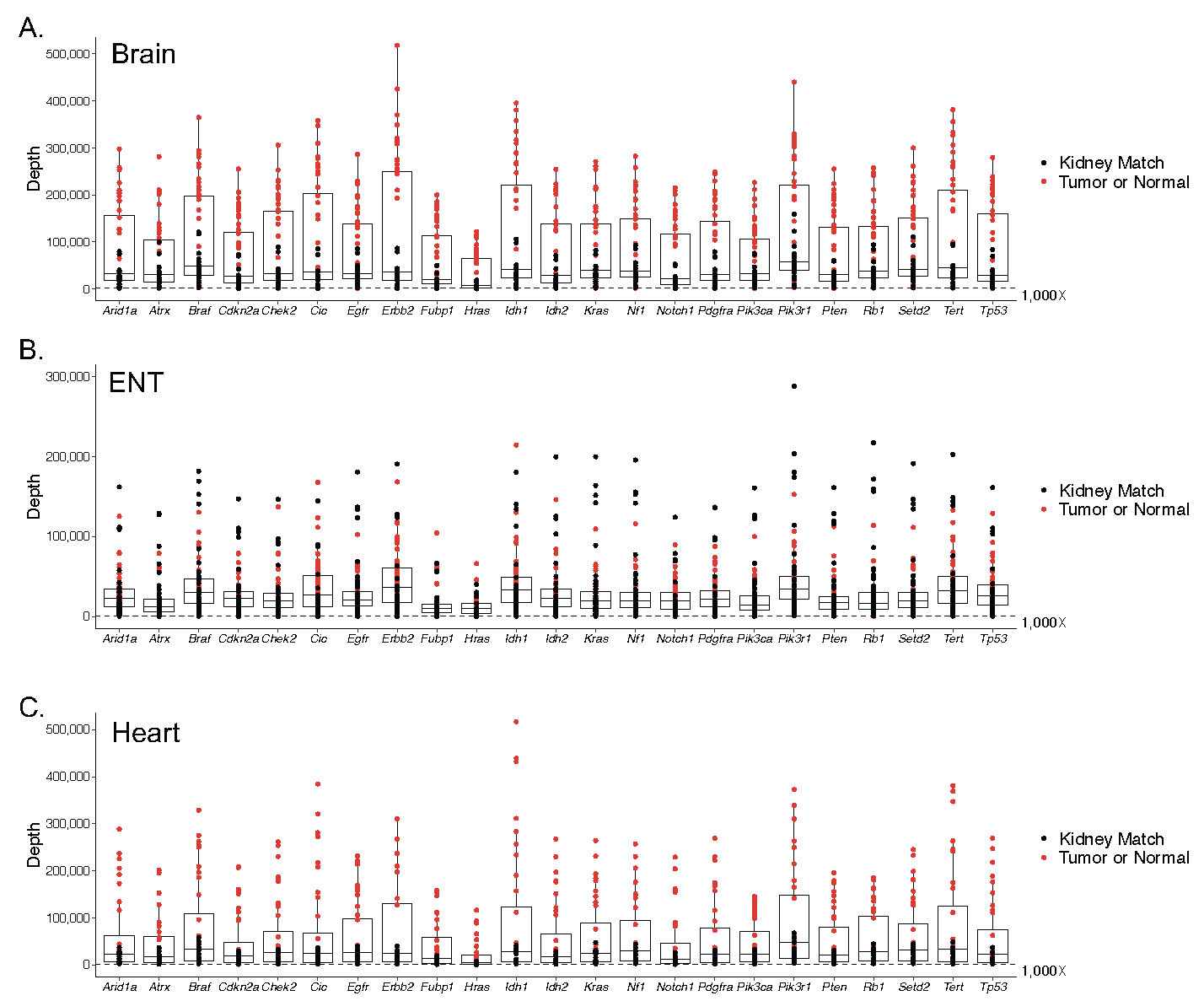

Supplement: S2 Fig — All the genes were sequenced to at least 1000x coverage in brain, ENT and heart samples with the exception of Hras that had a mean coverage of ~100x. (TIF) [file pone.0296699.s002.tif]
